# *In Vitro* Anthelmintic Activity of Crude Extracts of Aerial Parts of* Cissus quadrangularis* L. and Leaves of* Schinus molle* L. against* Haemonchus contortus*

**DOI:** 10.1155/2017/1905987

**Published:** 2017-12-19

**Authors:** Selamawit Zenebe, Teka Feyera, Solomon Assefa

**Affiliations:** ^1^Department of Veterinary Clinical Studies, College of Veterinary Medicine, Jigjiga University, Jigjiga, Ethiopia; ^2^Department of Pharmacology and Clinical Pharmacy, School of Pharmacy, Addis Ababa University, Addis Ababa, Ethiopia

## Abstract

**Background:**

* Haemonchus contortus, *the causative agent of Haemonchosis, is the most economically important parasite in small ruminant production. Control with chemotherapy has not been successful due to rapid emergence of drug-resistant strains. There is a continuous search for alternative leads particularly from plants. The study aimed to evaluate the anthelmintic activity of crude methanolic extracts of leaves of* Schinus molle *and aerial parts of* Cissus quadrangularis* against* H. contortus. Methods*. Adult motility test and egg hatching inhibition assay were employed to investigate the* in vitro *adulticidal and egg hatching inhibitory effects of the extracts.

**Results:**

Higher concentrations of the extracts (10 and 5 mg/ml) had a significantly superior adulticidal activity (*p* < 0.05) compared to the negative control and lower concentration levels, which was comparable to albendazole. Similarly, the relative egg hatch inhibition efficacy of* S. molle *and* C. quadrangularis *extracts indicated a maximum of 96% and 88% egg hatch inhibition, respectively, within the 48 hrs of exposure at 1 mg/ml.

**Conclusion:**

The current study evidenced that the crude methanolic extracts of the plants have promising adulticidal and egg hatching inhibitory effects against* H. contortus*.

## 1. Background

Parasitic infections remain a major constraint to livestock production globally [1].* Haemonchus contortus*, the causative agent of Haemonchosis, is a nematode parasite that feeds on blood of small ruminant animals and causes anaemia, anorexia, reduced growth, and eventual death of host animals [1, 2].* H. contortus *is highly pathogenic parasite of small ruminants and it is the primary constraint to profitable production of sheep and goats worldwide [2, 3].

Control is generally achieved by use of synthetic anthelmintics in combination with grazing management [4]. Synthetic anthelmintics have several drawbacks including resistance.* H. contortus* has been documented to be resistant to broad and narrow spectrum families of anthelminthics [5, 6]. Country-wide surveys for anthelmintic resistance have not yet been carried out in Ethiopia [7]. However, studies showed that resistance was detected in different parts of Ethiopia against albendazole, levamisole, tetramisole, and ivermectin [8].

One practical way of developing cheaper and effective anthelmintics is to study indigenous herbal remedies [9]. Evaluation of the activities of medicinal plants claimed for anthelmintic property is getting attention these days. There have been many reports, mainly from Africa, indicating the effectiveness of plant products against helminth infections in animals [10–12].

Studies reported that* Cissus quadrangularis* and* Schinus molle *are used against various helminth infections in Ethiopia*. C. quadrangularis *is claimed to be used in livestock against helminthiasis, tick and lice infestation, and leach infestation. Similarly,* S. molle *is widely used by pastoralists and agropastoralists of Ethiopia to eradicate intestinal parasites [13, 14]. Therefore, it was found necessary to evaluate the anthelmintic potential of two of the commonly used herbs by the pastoralist communities of Ethiopia. Thus, the present work was aimed at evaluating the* in vitro *egg antihelmintic efficacy of these plants against* H. contortus.*

## 2. Methods

### 2.1. Plant Collection and Extracts Preparation

Fresh aerial parts of* C. quadrangularis* and leaves of* S. molle* were collected from their natural habitat around Jigjiga, eastern Ethiopia. After botanical identification of the collected plants, voucher specimens, SZ01 for* C. quadrangularis* and SZ02 for* S. molle*, were deposited at the National Herbarium of Addis Ababa University, College of Natural Sciences. Plants were then cleaned, shade-dried, mechanically grinded, and coarsely powdered using laboratory mortar and pestle.

The crude extracts were prepared by cold maceration technique. Coarsely powdered plant materials were separately soaked in extraction solvent (methanol) followed by shaking periodically for three days and then filtered. The residue left after maceration was successively extracted twice with the same medium separately and the filtrate was passed through sterile filter paper (Whatman No. 3, Whatman Ltd., England). The filtrate was concentrated with rotary evaporator (Buchi Rota vapor, Switzerland). The extracts were then dried in hot air oven and transferred into well labeled vials and kept in a refrigerator until required for use. The resulting dry extract was weighed and provided a percentage yield of 5.3% (w/w) and 15.2% (w/w) for* C. quadrangularis* and* S. molle*, respectively.

### 2.2. Phytochemical Screening

Phytochemical screening was carried out to assess the qualitative chemical composition of crude methanolic extracts of* C. quadrangularis* and* S. molle. *Standard screening tests using conventional protocol, procedure, and reagents were conducted using standard procedures to identify the constituents as described in [15–17].

### 2.3. Collection of Parasites

Adult* H. Contortus *were collected from the abomasum of sheep obtained from Jigjiga municipal abattoir. Then, the abomasum was washed with water and the parasites were kept in phosphate buffer saline (PBS) until the* in vitro *evaluation was started.

### 2.4. *In Vitro *Anthelmintic Activity Evaluation

#### 2.4.1. Egg Hatch Inhibition Assay (EHIA)

Freshly collected adult female* H. contortus* were picked, crushed, and sieved to obtain the eggs, which were then triturated in PBS. The suspensions were centrifuged for 2 minutes at 300 rpm and sediment was retained. This sediment was resuspended in saturated solution of NaCl to form a convex meniscus above the test tube. After putting a coverslip above the test tube, samples were centrifuged again. Coverslip was carefully removed and eggs were washed into another test tube. This solution was then centrifuged and eggs were collected from sediment. Eggs were washed thrice with distilled water and adjusted to a concentration of 100–200 eggs/mL, using the McMaster technique [18].

EHIA was performed following the technique of Coles et al. [19]. Approximately, 100 eggs in 200 *μ*L of water were pipetted into each well of a 48-well microtiter plate. To each of the test wells, 200 *μ*L of each plant extract was added to a final volume of 400 *μ*L per well. The plant extracts were tested at concentrations of 0.1, 0.25, 0.5, and 1 mg/mL. Similarly, 200 *μ*L of albendazole (standard drug) at 0.25 mg/mL concentration and distilled water were used as a positive control and nontreated control, respectively.

Each test was done in three replicates. The plate was incubated in a humidified incubator at 37°C for 48 hrs. Thereafter, a drop of Lugol's solution was added to stop further hatching. All unhatched eggs and L1 larvae in each well were counted. The percent inhibition of egg hatching was calculated by using the formula below [19].

(1) $$\begin{array}{c} \text{Percent inhibition}\left(\;\%\;\right)=100\left(\;1-\frac{P_{test}}{P_{non\text{-}treated}}\;\right), \end{array}$$

where *P* is the number of eggs that hatched in EHIA.

#### 2.4.2. Adult Motility Assay (AMA)

AMA was conducted on mature* H. contortus* worms following the technique of Sharma et al. [20]. The test was performed in 5 cm diameter glass Petri dish. A total of about 368 adult parasites were used in the study. Four concentrations were employed for each plant extract. Ten worms were exposed in triplicate to each of the following treatments in separate Petri dishes at room temperature (25–30°C).

There were 4 groups as follows: Group I: crude methanol extract at 1.25, 2.5, 5, and 10 mg/mL of* C. quadrangularis* (four different concentrations prepared in PBS); Group II: crude methanol extract at 1.25, 2.5, 5, and 10 mg/ml of* S. molle*; Group III: albendazole at 0.25 mg/ml (positive control); and Group IV: PBS (negative control).

The inhibitions of motility of worms were used as indication of worm mortality or paralysis. Motility of worms was observed and motile worms were counted at different time intervals till 7 hrs posttreatment. Worms not showing any motility were picked out and kept in lukewarm PBS for 10 minutes and, in case of revival in motility, the observed worms were counted as alive; otherwise, they were counted as dead.

### 2.5. Data Analysis

Data were organized, edited, and analyzed using SPSS Version 20. The data obtained from both assays were analyzed with one-way ANOVA using Tukey HSD multiple comparison test. Results were deemed statistically significant if *p* < 0.05 at 95% confidence intervals.

## 3. Results

### 3.1. Phytochemical Screening

The phytochemical screening showed the presence of alkaloid and tannin in the both extracts whereas flavonoids and phenols were additionally present in the methanolic extract of* C. quadrangularis.*

### 3.2. Anthelmintic Activity

Both* in vitro *assays showed that crude extracts of both plants have promising adulticidal and egg hatching inhibitory effects. The extracts produced a dose-dependent anthelmintic activity in both AMA and EHIA.

#### 3.2.1. Adult Motility Test

This study indicated that both extracts produced a relatively comparable anthelmintic activity with the conventional anthelmintic, albendazole. The activity increased with concentration and time. After 7 hours of exposure of adult* H. contortus *to different concentrations of plant extracts, significant (*p* < 0.05) and dose-dependent reduction in motility was observed for both plants (Table 1).

**Figure 1:**
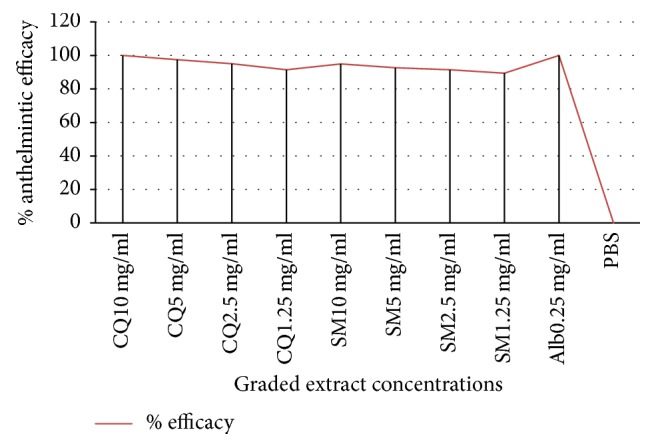
Relative adulticidal efficacy of graded concentration of crude extracts of C*. quadrangularis *and* S. molle. *CQ =* Cissus quadrangularis, *SM =* Schinus molle, *Alb = albendazole, and PBS = phosphate buffer saline.

At highest concentration (10 mg/mL), both plants produced mortality of adult* H. contorts *to the level of 95% and 100% after 7 hr exposure to the extracts, respectively (Figure 1). Albendazole, on the other hand killed the parasites in a time-dependent manner and all the adult worms were dead at a concentration of 0.25 mg/mL within 4 hrs after exposure.

The adulticidal efficacy profile of the extracts, as measured by the percentage of the adult parasites killed at the end of observation period, is as follows: 100 and 95% at concentration of 10 mg/ml, 97.5 and 92.6% at concentration of 5 mg/ml, 95.08 and 91.4% at concentration of 2.5 mg/ml, and 91.4 and 89.4% at concentration of 1.25 mg/mL for the* C*.* quadrangularis* and* S. molle*, respectively.

#### 3.2.2. Egg Hatching Inhibition Assay

The result of EHIA at graded concentration of crude methanolic extracts of* C. quadrangularis *and* S. molle *is shown in Table 2. The result indicated that both extracts produced a relatively comparable egg hatching inhibitory effect with albendazole. The methanolic extract of leaves of* S. molle* required a maximum concentration of 1 mg/ml to induce 96% egg hatch inhibition while* C. quadrangularis *induced 88% egg hatching inhibitory effect at the same concentration. The egg inhibitory efficacy profile of the extracts, as measured by the percentage of eggs unhatched at the end of observation period, is as follows: 40.67 and 39.33% at concentration of 0.1 mg/ml, 52 and 57% at concentration of 0.25 mg/mL, 70.33 and 74.33% at concentration of 0.5 mg/mL, and 88 and 96% at concentration of 1 mg/ml for the* C*.* quadrangularis* and* S. molle,* respectively.

## 4. Discussion

The problem of anthelmintic resistance, toxicity, and the increasing concern over the presence of drug residues in animal products has led to a renewal of interest in the use of plant based drugs. Plant materials evaluated in the current study had been identified from various sources to serve as anthelmintic agents by traditional healers of Ethiopia. The* in vitro *tests using free living stages of parasitic nematodes offer a means of evaluating the anthelmintic activity of new plant compounds [21].* In vitro* techniques are preferred to* in vivo* methods due to their low cost, simplicity, and rapid turnover [22]. In the current study, a statistically significant association was noted between graded concentrations of the extracts, the exposure test-time interval, and adult parasite mortality.

The whole plant of* C. quadrangularis *is documented to possess medicinal properties in ethnobotanical surveys conducted by ethnobotanists in traditional system of medicine [23]. Moreover, Luseba et al. [24] reported that the methanol extract and dichloromethane extract of stems of* C. quadrangularis* possess antimicrobial activity. The present study showed 100% efficacy of the plant extract against the parasite at the concentration of 10 mg/ml which was the highest efficacy value and was comparable with the standard anthelmintic, albendazole. The egg hatching inhibitory effect of this plant extract was 88% at the concentration of 1 mg/ml.

In the folk medicine,* S. molle *is an extensively studied medicinal plant throughout the world and has been reported to be used against wide ranges of human and livestock ailments [25–27]. In Somali Regional State of Ethiopia,* S. molle *is well used against endoparasites by pastoralists and agropastoralists. The leaves of* S. molle *are also reported to be used for the treatment of infections caused by different parasites [26]. This is evident from the current study, which showed 95% mortality of adult parasites of* H. contortus *at a concentration of 10 mg/ml in methanolic extracts of* S. molle*. The egg hatching inhibitory effect of* S. molle *was 96% at a concentration of 1 mg/ml. Increment on the concentration of the plant extracts resulted in increased inhibition of egg hatching indicating dose-dependent activity.

As screened in the phytochemical test of* S. molle, *the secondary metabolites, alkaloid and tannin, are responsible for their anthelmintic activity. In phytochemical screening of* C. quadrangularis*, it is revealed that the plant has secondary metabolites like alkaloids, flavonoids, tannins, and phenols. These classes of plant secondary metabolites are considered the sources of chemical components responsible for wide therapeutic activities of several medicinal plants [28]. Some studies are available for anthelmintic activity of tannins, alkaloids, and flavonoids [29, 30]. The presence of these phytochemicals may be responsible for the observed anthelmintic activity of plant extracts in the present study. Furthermore, tannins have been shown to interfere with coupled oxidative phosphorylation, thus blocking ATP synthesis in these parasites [31]. Finally, the* in vitro *methods provide a means to screen rapidly for potential anthelmintic activities of different plant extracts. Due to drug biotransformation, interaction with food materials, and absorption variations, the results obtained by the* in vitro* method could not be extrapolated to* in vivo* activity. Therefore, results should be ascertained by* in vivo *evaluation.

## 5. Conclusion

The current study evidenced that the methanolic extracts of aerial parts of* C. quadrangularis* and leaves of* S. molle *have a promising* in vitro *anthelmintic activity against adult and oval stages of* H. contortus. *However, the anthelmintic activity of* C. quadrangularis *was superior to* S. molle. *Fractionation of the crude extracts and further anthelmintic efficacy studies involving other parasite development stages are warranted.

## Figures and Tables

**Table 1:** *In vitro* adulticidal effect of crude extracts of *C. quadrangularis *and *S. molle *against *H. contortus.*

Treatment	Concentration	Number of parasites dead posttreatment (hours)						
		1 hr	2 hr	3 hr	4 hr	5 hr	6 hr	7 hr
*C. quadrangularis*	10 mg/mL	4.00 ± 0.58^acd^	5.33 ± 0.33^abcd^	7.33 ± 0.33^abcd^	8.67 ± 0.33^acd^	9.33 ± 0.33^acd^	10.00 ± 0.00^acd^	10.00 ± 0.00^acd^
	5 mg/mL	2.33 ± 0.33^abd^	3.67 ± 0.33^abcd^	4.33 ± 0.33^abcd^	5.00 ± 0.58^abd^	6.33 ± 0.33^abd^	7.33 ± 0.33^abcd^	8.67 ± 0.33^acd^
	2.5 mg/mL	0.67 ± 0.33^b^	2.00 ± 0.00^ab^	3.33 ± 0.33^abd^	4.67 ± 0.33^ab^	6.33 ± 0.33^abd^	7.00 ± 0.58^abd^	7.33 ± 0.33^abcd^
	1.25 mg/mL	0.67 ± 0.33^b^	1.67 ± 0.33^b^	2.67 ± 0.33^ab^	3.67 ± 0.33^ab^	4.67 ± 0.33^ab^	5.33 ± 0.33^ab^	5.33 ± 0.33^ab^
*S. molle*	10 mg/mL	2.00 ± 0.58^abd^	3.00 ± 0.58^abd^	4.33 ± 0.33^abcd^	6.00 ± 0.58^abcd^	6.67 ± 0.66^abcd^	7.00 ± 0.58^abd^	7.33 ± 0.33^abcd^
	5 mg/mL	0.67 ± 0.33^b^	1.33 ± 0.33^b^	2.33 ± 0.33^ab^	3.33 ± 0.33^ab^	3.67 ± 0.33^ab^	5.33 ± 0.33^ab^	6.00 ± 0.00^abd^
	2.5 mg/mL	0.33 ± 0.33^b^	1.33 ± 0.33^b^	2.33 ± 0.33^ab^	4.67 ± 0.33^ab^	4.67 ± 0.33^ab^	5.00 ± 0.00^ab^	5.33 ± 0.33^ab^
	1.25 mg/mL	0.00 ± 0.00^b^	0.67 ± 0.33^b^	1.67 ± 0.33^ab^	3.00 ± 0.58^ab^	3 ± 0.58^ab^	3.67 ± 0.33^ab^	4.33 ± 0.33^ab^
Albendazole	0.25 mg/mL	5.33 ± 0.33^acd^	8.33 ± 0.33^acd^	9.33 ± 0.33^acd^	10.00 ± 0.00^acd^	10.00 ± 0.00^acd^	10.00 ± 0.00^acd^	10 ± 0.00^acd^
PBS	1 mL	0.00 ± 0.00^b^	0.00 ± 0.00^b^	0.00 ± 0.00^bcd^	0.67 ± 0.33^bcd^	0.67 ± 0.33^bcd^	1.33 ± 0.33^bcd^	2.67 ± 0.33^bcd^

Values are mean ± *SEM*. All superscripts indicate significance at *p* < 0.05, ^a^compared to untreated (PBS), ^b^compared to albendazole, ^c^compared to lowest dose of methanolic extract of *C. quadrangularis*, and ^d^compared to lowest dose of methanolic extracts of *S. molle*.

**Table 2:** *In vitro* EHIA effect of crude extracts of *C. Quadrangularis *and *S. molle *against *H. contortus, *48 hours posttreatment.

Treatment	Concentration	Number of unhatched eggs	Number of L1 larvae	Percentage of inhibition
*C. quadrangularis*	0.1** **mg/mL	54.3 ± 33.06^ab^	45.67 ± 3.05^abd^	40.67 ± 1.53^ab^
	0.25** **mg/mL	66.00 ± 2.00^abd^	37.00 ± 2.00^abd^	52.00 ± 4.35^abd^
	0.5** **mg/mL	77.00 ± 2.64^acd^	23.00 ± 2.64^acd^	70.33 ± 4.04^abcd^
	1** **mg/mL	89.00 ± 3.60^abc^	11.00 ± 3.60^abc^	88.00 ± 5.29^abcd^
*S. molle*	1** **mg/mL	97.00 ± 2.00^abc^	3.00 ± 2.00^abc^	96.00 ± 2.52^abc^
	0.5** **mg/mL	43.00 ± 3.00^abc^	57.00 ± 3.00^abc^	74.33 ± 3.22^abcd^
	0.25** **mg/mL	56.00 ± 1.00^abc^	44.00 ± 1.00^abcd^	57.00 ± 3.60^abcd^
	0.1** **mg/mL	69.33 ± 4.50^abcd^	30.67 ± 4.50^abcd^	39.33 ± 3.78^ab^
Albendazole	0.25** **mg/mL	98.33 ± 5.50^acd^	1.67 ± 5.50^cd^	99.67 ± 5.50^acd^
Distilled water		94.00 ± 2.00^cd^	6.00 ± 2.00^cd^	7.33 ± 2.08^bcd^

Values are *mean *±* SEM*. All superscripts indicate significance at *p* < 0.05, ^a^compared to untreated (distilled water), ^b^compared to albendazole, ^c^compared to lowest dose of methanolic extract of *C. quadrangularis*, and ^d^compared to lowest dose of methanolic extracts of *S. molle*.

## Abbreviations

AMA: Adult motility assay
EHIA: Egg hatch inhibition assay
PBS: Phosphate buffer saline
NaCl: Sodium chloride.

## Data Access

Vouchers and dried leaves used for this study are stored at the herbarium at the Addis Ababa University, College of Natural Sciences. The datasets supporting the conclusion of this study are available from the corresponding author on reasonable request.
